# Food allergy and attention-deficit/hyperactivity disorder in children: evidence and implications for pediatric allergy practice

**DOI:** 10.3389/fped.2026.1982325

**Published:** 2026-10-16

**Authors:** Bingxue Lu, Yongmei Xie

**Affiliations:** 1 Department of Pediatric Gastroenterology, West China Second University Hospital of Sichuan University, Chengdu, China; 2 Key Laboratory of Birth Defects and Related Diseases of Women and Children, Ministry of Education, Sichuan University, Chengdu, China; 3 West China College of Clinical Medicine, Sichuan University, Chengdu, China

**Keywords:** attention-deficit/Hyperactivity disorder, avoidant/restrictive food intake disorder, elimination diet, food allergy, gut–brain axis, integrated care, neurodevelopment, pediatric allergy

## Abstract

Families of children with food allergies (FA) and attention-deficit/hyperactivity disorder (ADHD) often ask whether the conditions are related and whether dietary changes could improve their children's attention or behavior. This narrative review examines FA-specific evidence and its implications for pediatric allergy practices, drawing on observational studies, reviews, clinical guidance, and contextual mechanistic research. One small FA-specific meta-analysis found no statistically significant association, whereas several individual observational studies reported positive associations. Differences in diagnostic definitions, reporting sources, temporal assessment, and confounder adjustment limited the ability to make comparisons. Studies of broader allergic phenotypes and Mendelian randomization provide no FA-specific causal estimate, and proposed microbiota–immune–brain pathways remain unconfirmed in humans. Restrictive or elimination diets should not be routinely recommended for ADHD because evidence of sustained benefit is limited, and additional restriction may compromise nutrition or worsen feeding difficulties, particularly in children already avoiding allergens. Pediatric allergy care should verify the FA diagnosis, preserve tolerated foods, monitor growth and dietary adequacy, and refer persistent attention or behavioral concerns for appropriate assessment. This review distinguishes direct FA evidence from broader allergy findings and relates these uncertainties to practical decisions about diagnosis, dietary restriction, and coordinated care.

## Introduction

Attention-deficit/hyperactivity disorder (ADHD) is a heterogeneous neurodevelopmental condition characterized by developmentally inappropriate inattention and/or hyperactivity–impulsivity, with functional impairment across settings (1). Its clinical assessment requires attention to developmental history, impairment, and other conditions that can affect behavior. In pediatric allergy practices, families may ask whether food-related symptoms or dietary restriction contribute to their children's attention difficulties and whether changing the diet could improve ADHD.

A food allergy (FA) is an immune-mediated adverse reaction to food. For suspected IgE-mediated FA, diagnosis depends on interpreting a compatible medical history alongside sensitization testing, with a supervised oral food challenge used when uncertainty remains (2). FA and ADHD can disrupt eating, sleep, school participation, and family routines. Anxiety about reactions, sleep disturbances, restrictive eating, and proposed immune or gut microbiota pathways offer possible connections, but clinical burden and mechanistic plausibility do not prove that FA causes ADHD.

The existing evidence is difficult to interpret because the definitions of FA and ADHD, the reporting sources, the time frames, and the covariate adjustments differ across studies. Sensitization, confirmed FA, and composite allergic disease are not interchangeable exposures. Co-occurrence must also be distinguished from temporal sequence and causation. This review aims to critically synthesize evidence most directly relevant to FA and ADHD, distinguish it from broader allergy and mechanistic evidence, and identify its implications for diagnosis, nutrition, and coordinated pediatric care.

## Materials and methods

### Review scope and literature search

This focused narrative review drew on a PubMed search covering literature published up to 28 August 2026. During revision, six reproducible searches were conducted on 30 September 2026, using the same publication date limit. The searches addressed direct FA–ADHD evidence and relevant literature on broader allergic diseases, feeding and psychosocial outcomes, microbiota-related mechanisms, FA diagnosis and nutritional management, and ADHD diet and treatment. Sources were selected based on their relevance to the review question and pediatric allergy practice. The exact search strategies and retrieval counts are provided in Supplementary Table S1. No systematic review protocol or meta-analysis was undertaken.

### Literature selection and evidence synthesis

Bingxue Lu selected the literature and drafted the synthesis; Yongmei Xie reviewed the manuscript and its interpretation. Selection and data extraction were not performed independently in duplicate. Human studies reporting FA separately in relation to ADHD diagnoses or symptoms form the core evidence of this review. Studies of composite allergic diseases, Mendelian randomization of broader allergic phenotypes, and animal or microbiome research provide context. Clinical guidance and dietary intervention studies inform the discussion on clinical practices. Findings on autism, anxiety, sleep, or feeding are discussed where relevant to clinical interpretation, with these outcomes distinguished from ADHD. The scope describes the narrative literature selection and does not constitute a set of prospectively registered eligibility criteria.

For the present synthesis, study design, sample information, phenotype ascertainment, reported estimates, and interpretive limitations were organized in Table 1. FA-specific and composite-allergy estimates were kept separate, and adjusted and unadjusted results were distinguished. Evidence was synthesized narratively according to consistency, temporal sequence, phenotype specificity, and alternative explanations. No standardized risk-of-bias tool or formal certainty framework was applied. The separate qualitative appraisal column is a descriptive assessment of the listed methodological limitations, not a validated score or a GRADE rating. Certainty judgments attributed to a published review are identified as that review's findings.

**Table 1:** Human evidence informing the food allergy–ADHD question.

Study and design	Phenotype ascertainment	Main estimate	Qualitative appraisal	Interpretation and limitations
A. FA-specific estimates				
Miyazaki et al. (3) Systematic review; 3 FA studies	The definitions of FA and ADHD varied across the included studies.	OR 1.13 (95% CI 0.88–1.47); certainty very low.	Very low certainty as rated by the source review	The pooled estimate was not significant, but it rests on only three heterogeneous FA studies.
Ferro et al. (4) Prospective cohort dataset; *n* = 1,303	FA status; maternal- and self-reported ADHD symptom measures.	Maternal report of elevated ADHD symptoms in adolescence: OR 3.14 (95% CI 1.07–9.19); self-report not significant.	Limited by reporter dependence and imprecision	The association appeared only in maternal reports; the wide CI and symptom-based outcome limit interpretation.
Shanahan et al. (5) Longitudinal community cohort; *n* = 1,420; 5,165 interview pairs	Parent-reported food or digestive allergies in the previous year; structured psychiatric interviews; ADHD symptoms and DSM-IV diagnoses.	ADHD symptom mean ratio: 1.88 (95% CI 1.18–2.99) cross-sectionally; 0.95 (0.54–1.67) longitudinally. Cross-sectional ADHD diagnosis OR 2.39 (0.93–6.13); diagnosis associations not significant.	Limited by reported FA and imprecise diagnosis estimates	Concurrent symptoms differed, but prospective ADHD symptom change and ADHD diagnoses were not significantly associated. Age and sex were adjusted; longitudinal symptoms were also adjusted for prior symptoms.
Jiang et al. (6) Cross-sectional survey; *n* = 22,018	Parent-reported early FA symptoms and ADHD history.	Adjusted OR 1.53 (95% CI 1.13–2.05) for FA without respiratory allergy; higher with a coexisting respiratory allergy.	Limited by recall and temporal uncertainty	The sample size was large, but retrospective parent reports left recall, classification, and temporal uncertainty.
Xu et al. (7) Pooled US NHIS surveys; *n* = 192,573	Adult-reported food or digestive allergy in the past year and lifetime health professional ADHD diagnosis.	Adjusted OR 1.72 (95% CI 1.55–1.91); 1.44 (1.29–1.60) after adjustment for other allergies.	Limited by reported phenotypes and cross-sectional ascertainment	Adjustment for other allergies weakened the estimate; the cross-sectional design could not establish sequence.
Yang et al. (8) Cross-sectional preschool survey; *n* = 2,772; 28 ADHD cases	Questionnaire-reported physician-diagnosed FA plus compatible symptoms; physician-diagnosed ADHD; stratified by skin-prick sensitization.	Adjusted OR: 1.94 (95% CI 0.44–8.46) without sensitization; 2.01 (0.59–6.90) with sensitization; neither was significant.	Limited by sparse ADHD cases and wide confidence intervals	Adjusted for age, sex, maternal atopy, birth weight, maternal education, and breastfeeding. Sensitization was to any tested allergen, not necessarily food. Cross-sectional data did not establish sequence.
Nemet et al. (9) Retrospective cohort; *n* = 234,170 (8,174 with FA)	Allergist-diagnosed FA and clinically coded ADHD.	Unadjusted FA–ADHD OR 1.59 (95% CI 1.45–1.70); no FA-specific adjusted estimate.	Limited by absent FA-specific confounder adjustment	FA was recorded more specifically, but the FA estimate remained unadjusted; visit-frequency adjustment was applied only to the composite exposure.
Singer et al. (10) Retrospective primary-care EMR cohort; *n* = 288,490 (7,312 with FA)	Physician-recorded FA was identified from electronic medical records; documented ADHD comorbidity.	Age- and sex-adjusted OR 1.81 (95% CI 1.66–1.96).	Limited by record-based ascertainment and residual confounding	Physician recording differed from caregiver-only report but did not imply a uniform, challenge-confirmed FA. The reported ADHD model was adjusted for age and sex; the association did not establish sequence or causation.
Husain et al. (11) Cross-sectional ALSPAC birth-cohort analysis; research letter	Parent-reported FA from age 7; likely ADHD at age 9, as defined by the SDQ hyperactivity scale.	Sex-adjusted OR 1.82 (95% CI 1.23–2.69) for FA among children with likely ADHD.	Limited by screening-defined ADHD, reported FA, and sex-only adjustment	The model treated FA as the outcome. Parent reports may include intolerance or aversion; SDQ classification is not an ADHD diagnosis. Temporal sequence and causation remain unresolved.
B. Indirect or composite allergy evidence				
Zhang et al. (14), with 2026 commentary (15) Two-sample Mendelian randomization	Genetically predicted ADHD and five allergic diseases; none was FA.	Allergic asthma: IVW OR 1.06 (95% CI 1.02–1.10); alternative estimators and stricter instrument selection not significant.	Indirect for FA; estimates vary by MR method	Estimates differed across MR methods, and later commentary raised methodological concerns; no FA-specific causal estimate was tested.
Liu et al. (16) Bidirectional two-sample Mendelian randomization	ADHD genetic liability and a broad childhood allergy phenotype; not FA.	ADHD liability→childhood allergy: OR 1.14 (95% CI 1.02–1.27); no microbiota/metabolite mediation.	Indirect for FA; broad allergy phenotype	The tested direction was ADHD liability toward broad childhood allergy, not FA as an exposure for ADHD.
Çelik et al. (12) Retrospective cohort; *n* = 860,089	Composite ‘other allergies' (food/drug allergy and/or anaphylaxis); 619 cases had FA; no FA-specific estimate.	Composite other-allergy RR 1.30 (95% CI 1.16–1.46) in the reported incidence analysis; no FA-specific estimate.	Indirect for FA; composite exposure and limited adjustment	The cohort was large, but the composite exposure and limited adjustment did not permit an FA-specific interpretation.

FA-specific means that FA was analyzed separately; it does not imply clinical confirmation in every study. Qualitative appraisal describes the stated limitations without a standardized score. The very low certainty label for Miyazaki et al. is taken from the source review and was not assigned in this narrative review. ADHD, attention-deficit/hyperactivity disorder; ALSPAC, Avon Longitudinal Study of Parents and Children; CI, confidence interval; EMR, electronic medical record; FA, food allergy; IVW, inverse-variance weighted; MR, Mendelian randomization; NHIS, National Health Interview Survey; OR, odds ratio; RR, relative risk; SDQ, Strengths and Difficulties Questionnaire.

FA was not consistently ascertained across studies. Many epidemiological reports rely on parent or caregiver reports, and the majority of these do not distinguish IgE-mediated from non-IgE-mediated disease. We therefore retained the source study's definition of FA and indicated, when possible, whether the phenotype was clinically established or simply reported. Recommendations on skin-prick testing, food-specific IgE, and oral food challenges primarily related to suspected IgE-mediated FA. Nutritional and feeding considerations applied more broadly whenever a medically necessary restricted diet is in place.

## Epidemiological evidence

### FA-specific evidence

FA-specific findings are inconsistent (Table 1). A systematic review by Miyazaki et al. found no statistically significant pooled association and rated the evidence as having very low certainty (3). In a cohort-based analysis, Ferro et al. found an association with maternally reported ADHD symptoms, but not with self-reported symptoms (4). The difference between reporters, the wide confidence interval, and the symptom-based outcome makes this finding difficult to generalize to clinically diagnosed ADHD. In the Great Smoky Mountains Study, reported food or digestive allergies were associated with more concurrent ADHD symptoms, but not with subsequent symptom increases or ADHD diagnoses (5). This distinction limits the interpretation of these associations as evidence of a persistent disorder.

Positive associations have also been reported in large questionnaire datasets. A Chinese school-age survey found an association between parent-reported early FA symptoms and ADHD history (6). In the U.S. National Health Interview Survey, the exposure was a reported food or digestive allergy in the preceding year, rather than clinically confirmed FA; the association with reported ADHD diagnoses weakened after adjustment for respiratory and skin allergies (7). Large samples improve statistical precision, but shared reporting sources, uncertain temporal sequence, and exposure misclassification remain important limitations. A Taiwanese preschool survey found no statistically significant FA–ADHD association in either sensitization stratum; few ADHD cases and wide confidence intervals limited precision (8).

Clinical databases provide more specific diagnostic recordings, but they do not eliminate confounding. An Israeli retrospective cohort study reported a positive unadjusted FA–ADHD association; healthcare visit frequency and other covariates were only adjusted for the composite allergy exposure, leaving no adjusted FA-specific estimate (9). A Canadian primary care database study found an association between physician-recorded FA and ADHD after adjustment for age and sex, with residual confounding still possible (10). A UK birth cohort analysis also reported an association between parent-reported FA and screening-defined likely ADHD, with adjustment limited to sex; neither measure established a clinically confirmed phenotype (11). A Turkish cohort study grouped FA with drug allergy and anaphylaxis and reported an association for that composite category even when allergy preceded ADHD ascertainment (12). Temporal restriction improved sequence assessment; however, the mixed exposure and limited confounder control prevented an FA-specific interpretation.

### Evidence from other allergic phenotypes

An umbrella review reported associations between allergic diseases and neurodevelopmental outcomes, with substantial variation in effect size and certainty across phenotypes (13). These findings require attention to both the allergic exposure and the behavioral outcome. Asthma, allergic rhinitis, and atopic dermatitis differ from FA in their symptoms and treatment. ADHD, autism spectrum disorder, anxiety, sleep disturbance, and avoidant/restrictive food intake disorder (ARFID) are also distinct clinical constructs, despite possible coexistence.

Pruritus, nasal obstruction, and nocturnal respiratory symptoms may affect sleep and daytime behavior in children with other atopic diseases. FA involves a different combination of episodic reactions, fear of exposure, and dietary avoidance. These differences affect the plausible mediators and confounders in each setting. Therefore, estimates for composite allergic disease provide context for the FA–ADHD question, but they cannot identify an FA-specific effect or mechanism (3, 7, 12, 13).

Mendelian randomization studies have examined ADHD genetic liability in relation to broader allergic outcomes. One study reported a small association with allergic asthma in its primary model, but this was not supported by alternative estimators or a stricter instrument-selection threshold (14). A subsequent commentary discussed concerns about heterogeneity reporting, instrument selection, and statistical power (15). A separate bidirectional analysis reported an association between ADHD liability and a broad childhood allergy phenotype, without evidence of mediation by the tested microbiota or metabolites (16). Neither study analyzed FA as a distinct phenotype (Table 1).

Several explanations for the observational association therefore remain plausible. A true relationship cannot be excluded, nor can surveillance effects, residual familial or socioeconomic confounding factors, coexisting atopic diseases, misclassified phenotypes, or reverse causation. Children who are observed repeatedly for FA have more opportunities for behavioral concerns to be noticed; similarly, ADHD may lead to more healthcare encounters, dietary concerns, and caregiver reporting. Sleep disruption, anxiety, and restrictive eating can also impair everyday attention. Existing designs do not establish whether these factors precede the association, lie on a causal pathway, or arise after one of the conditions develops.

## Clinical and mechanistic considerations

### Clinical burden and attention

FA can impose psychological and practical burdens through vigilance about accidental exposure, restrictions at school, and fear of reactions. A voluntary patient and caregiver registry reported frequent FA-related mental health concerns, particularly anxiety and fear; its self-selected sample should not be used to estimate population prevalence (17). A preschool case-control study also reported more emotional reactivity, somatic complaints, and sleep problems in children with FA, with behavioral difficulties related to maternal anxiety and perceived child vulnerability (18). These experiences may impair concentration or increase restlessness, but they are not equivalent to the persistent, cross-setting syndrome required for an ADHD diagnosis.

Clinical influence may also run in the opposite direction. Difficulties with planning, inhibition, or sustained attention can complicate allergen avoidance, emergency preparedness, and adherence. Selective eating and sensory sensitivity may narrow an already limited diet. One controlled study found higher ARFID screening scores in children with physician-diagnosed FA, particularly in the fear and picky eating domains (19). Children with ADHD have separately been reported to show more selective eating and sensory-related ARFID features than their peers (20). These findings concern feeding symptoms and screening profiles rather than proof of an ARFID diagnosis or a shared FA–ADHD etiology.

### Preclinical microbiota–immune–brain evidence

In an ovalbumin-induced FA mouse model, attention-related behavioral changes were accompanied by differences in prefrontal inflammatory markers, gut microbial composition, and serum metabolites (21). A subsequent experiment from the same group reported improvements in several behavioral measures after a multi-strain probiotic intervention (22). These models suggest mechanisms for further investigation; however, attention tasks in mice do not establish ADHD. Reduced intake, illness, stress, and immune activation may also influence the measured behaviors. Human studies are needed to determine whether the proposed pathways contribute to attention difficulties in children with FA.

Human microbiome evidence remains inconclusive. Reviews of ADHD studies describe inconsistent taxon-level findings and substantial variation in diet, medication exposure, sequencing, and analysis (23–25). Pediatric FA studies also report microbiome differences (26). Findings from these separate literatures do not demonstrate that the microbiome connects the disorders. No reproducible microbial biomarker or microbiome-directed treatment has been validated for children with both FA and ADHD.

## Implications for pediatric allergy practice

### Diagnostic assessment

The assessment of suspected FA should distinguish immune-mediated reactions from intolerance, non-specific gastrointestinal symptoms, and sensitization without clinical reactivity. The patient's history should clarify the timing, reproducibility, dose, symptom pattern, and treatment of the suspected reactions. Skin-prick and food-specific IgE results require interpretation in this clinical context. A supervised oral food challenge may resolve uncertainty where appropriate (2, 27, 28). Long-term avoidance should be reviewed when a diagnosis has not been established.

When ADHD is suspected, the assessment should establish clinically significant symptoms and impairment in more than one setting, confirm that several symptoms were present before the age of 12, and consider alternative explanations (1, 29). Pain, pruritus, nocturnal symptoms, anxiety, learning difficulties, inadequate nutrition, and insufficient sleep can affect attention or behavior. Laboratory investigation of nutritional deficiencies should follow a medical history and physical examination rather than being part of routine ADHD screening. These contributors may need treatment alongside, or independently of, ADHD.

### Nutrition and feeding

Medically necessary avoidance must support normal growth and an adequate diet. Guidance for cow's milk allergy emphasizes suitable substitutes and monitoring of intake and growth, particularly ensuring adequate calcium and vitamin D when dairy is excluded (30). Broader pediatric FA guidance supports reviewing energy and protein intake, the range of accepted foods, and other nutrients according to the foods removed and the clinical picture (31). Dietitian input is especially useful when several foods are excluded, growth falters, or when ADHD medication reduces appetite.

A feeding assessment should consider the reasons for avoidance, including fear after a reaction, sensory aversion, low appetite, gastrointestinal discomfort, and parental restriction. Progressive loss of accepted foods, persistent fear, nutritional compromise, mealtime conflict, or impaired social participation warrants assessment for a feeding disorder or ARFID (19, 20). Screening scores alone do not establish a diagnosis. Foods confirmed to be tolerated should be reintroduced with appropriate clinical and dietary support.

### Dietary restrictions and ADHD management

Avoiding a confirmed allergen is the treatment for FA. Restrictive or elimination diets should not be routinely recommended to manage ADHD. Some dietary studies reported symptom improvement, but small or selected samples, heterogeneous interventions, limited blinding, and uncertain durability prevented a favorable benefit–risk balance from being established for routine care (32–35). The TRACE follow-up study found less improvement and poorer adherence to the elimination diet than to the structured healthy diet trajectory; additional care and changes after the initial phase complicate interpretation (33). Retrospective few-foods diet monitoring had no randomized comparator (34). Food-color challenge studies addressed a different exposure and do not demonstrate that clinically confirmed FA cause ADHD (35).

Additional restrictions may reduce energy and nutrient intake, worsen selective eating or mealtime difficulties, and reinforce fear-based avoidance. It may contribute to the development or persistence of ARFID, although the cited studies do not measure the incidence of ARFID caused by ADHD diets (19, 20, 30, 31). Children who are already avoiding foods due to FA are particularly vulnerable to further restriction of their diet. Therefore, clinical reviews should assess nutrition and feeding, preserve tolerated foods, and provide established ADHD care (29). If a restrictive intervention is considered in an exceptional case, specialist and dietitian supervision, growth and nutritional monitoring, planned reintroduction, and stopping criteria are necessary. These precautions address potential harm, but evidence for routine use remains insufficient.

There is also a lack of sufficient evidence for the routine use of probiotics in ADHD management. Although an FA mouse experiment was mechanistically interesting (22), a recent synthesis of pediatric randomized trials found no clear overall ADHD-related benefit, with exploratory parent-reported signals varying across instruments (36). Evidence from general ADHD samples should not be interpreted as treatment evidence for children with both FA and ADHD.

### Coordinated care and follow-up

ADHD treatment should be selected according to age, functional impairment, expected benefit, adverse effects, and family preferences (29, 37). Evaluation of a suspected food-related contributor should not delay indicated treatment. Follow-up during medication use should include monitoring appetite, weight, growth, and other relevant adverse effects. Appetite suppression with stimulants is especially relevant when FA already limits dietary intake (29, 37).

Figure 1 outlines a coordinated approach to care. Allergy clinicians can clarify the FA phenotype, treat allergic symptoms, review diet and growth, and identify concerns requiring further assessment. General pediatric, developmental-behavioral, or child mental health clinicians can assess ADHD according to their training and local care pathways. Dietitians can advise on nutritional substitutes, and feeding or psychological support may be needed for persistent avoidance, disproportionate fear, or suspected ARFID. Follow-up should document allergic reactions, dietary adequacy, and growth alongside ADHD symptoms and functioning across settings. Improvement after treatment for either condition may reflect a reduced clinical burden but does not, by itself, establish a causal relationship.

**Figure 1:**
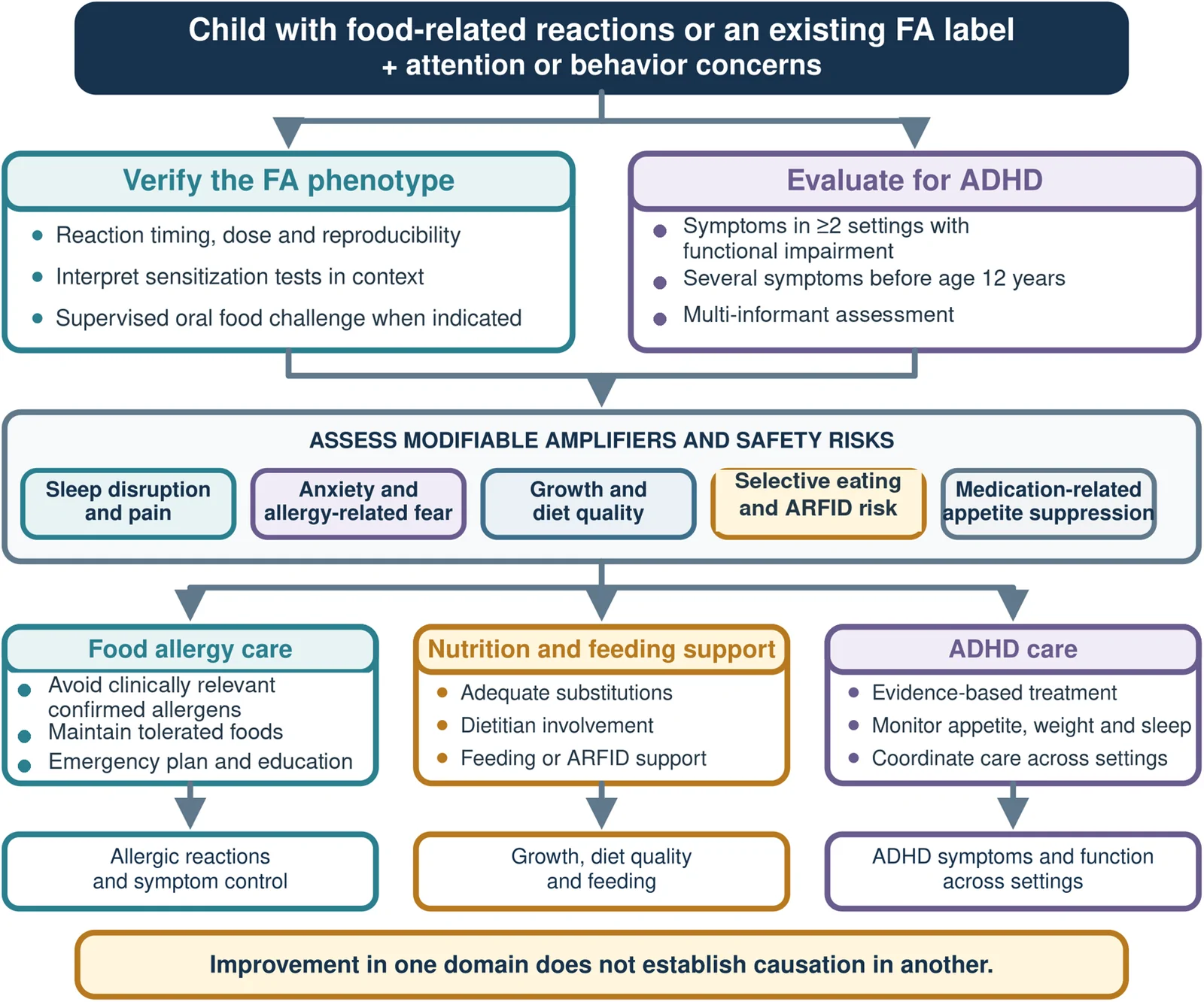
Clinical pathway for children seen in pediatric allergy practice with suspected or confirmed food allergies and concurrent attention or behavior concerns. FA and ADHD are evaluated on separate clinical tracks, while nutrition, feeding, sleep, and psychosocial factors are addressed when they contribute to functional difficulty. Changes in one domain should not be taken as evidence that they caused changes in the other. FA, food allergy; ADHD, attention-deficit/hyperactivity disorder; ARFID, avoidant/restrictive food intake disorder; OFC, oral food challenge.

## Discussion and research priorities

The available evidence supports assessment of persistent attention, school, sleep, and feeding problems in children with FA. However, it does not establish a basis for routine ADHD screening solely because FA is present, or for food exclusion solely because ADHD is diagnosed. Sleep disturbances, anxiety, restricted intake of food, and coexisting atopic diseases may contribute to functional difficulties in individual children. At the population level, shared reporting, healthcare utilization, residual confounding factors, and phenotype misclassification may also influence the observed associations. These clinical and methodological explanations should be considered when discussing dietary changes with families.

Prospective research needs more specific phenotype definitions and clearer temporal assessments. FA should, where feasible, be clinically established before subsequent neurodevelopmental assessment. ADHD outcomes should use validated information from more than one setting. Studies should distinguish between IgE-mediated FA and non-IgE-mediated gastrointestinal allergy, and between sensitization, intolerance, and other atopic diseases. Covariate selection should reflect an explicit causal model: familial and socioeconomic factors and healthcare utilization may confound an association, whereas sleep, anxiety, feeding restriction, and treatment exposure may also lie on pathways between symptoms and functioning. Prespecified causal diagrams would help clarify these assumptions and reduce inappropriate adjustment.

Sibling comparisons and negative controls could help assess shared familial factors and ascertainment effects. Repeated measurements of diet, growth, sleep, and behavior would improve the assessment of changes following avoidance or treatment. Linking clinical allergy records with prospective behavioral assessments would reduce reliance on caregiver recall. Mechanistic studies should establish the temporal sequence of the proposed mediators and replicate the findings in independent populations. Where justified, interventions should address a defined clinical problem, such as sleep disruption, nutritional deficiency, or ARFID, and evaluate attention and functioning with validated measures.

## Conclusion

Some observational studies report more ADHD diagnoses or symptoms among children with FA, but current evidence does not establish a consistent FA-specific association or demonstrate causation. Phenotype misclassification, uncertain temporal sequence, confounding factors, and indirect mechanistic evidence limit inference. Pediatric allergy care should confirm FA, maintain tolerated foods, protect growth and feeding development, and recognize persistent attention, sleep, or behavioral concerns that merit assessment. Restrictive or elimination diets should not be routinely recommended for ADHD management. FA and ADHD require evidence-based care for each condition, with coordinated support when their clinical burdens interact.
