# Supplementary material for: Food allergy and attention-deficit/hyperactivity disorder in children: evidence and implications for pediatric allergy practice
Source: Front Pediatr. 2026 Oct 16;14:1982325. doi: 10.3389/fped.2026.1982325 (PMC13720248; doi:10.3389/fped.2026.1982325)
Supplement: Supplementary file 1 [file Table1.docx]

Supplementary Table S1 PubMed searches performed during revision

Database: PubMed. Searches executed: 30 September 2026. Publication-date limit: 1 January 1900 to 28 August 2026.

These searches were performed during revision to document the search approach and check literature coverage. They are not the original search logs. The review remains a focused narrative Mini Review.

| **ID** | **Evidence domain** | **Records** |
| --- | --- | --- |
| S1 | Direct FA–ADHD evidence | 121 |
| S2 | Broader allergy–ADHD / causal context | 779 |
| S3 | Feeding / ARFID / psychosocial | 337 |
| S4 | Microbiota / immune / brain mechanism | 400 |
| S5 | FA diagnosis / nutrition / management | 620 |
| S6 | ADHD dietary treatment / probiotics / guideline | 423 |

The six exports contained 2,680 records, including 171 duplicate occurrences across searches. De-duplication by PMID yielded 2,509 unique records. Per-search counts include records retrieved by more than one search.

Thirty-two of the 37 cited sources were present in these exports. Five cited sources were retained as supplementary sources. This count describes source retrieval and citation selection, not a PRISMA systematic-review flow.

# Exact search strings

## S1 Direct FA–ADHD evidence

( "Food Hypersensitivity"[Mesh] OR "food allergy"[tiab] OR "food allergies"[tiab] OR "food hypersensitivity"[tiab] OR "food hypersensitivities"[tiab] ) AND ( "Attention Deficit Disorder with Hyperactivity"[Mesh] OR ADHD[tiab] OR "attention-deficit/hyperactivity disorder"[tiab] OR "attention deficit hyperactivity disorder"[tiab] OR "attention deficit disorder with hyperactivity"[tiab] ) AND ("1900/01/01"[dp] : "2026/08/28"[dp])

## S2 Broader allergy–ADHD / causal context

( allerg*[tiab] OR asthma[tiab] OR "allergic asthma"[tiab] OR "allergic rhinitis"[tiab] OR "atopic dermatitis"[tiab] OR eczema[tiab] OR "childhood allergy"[tiab] ) AND ( ADHD[tiab] OR "attention-deficit/hyperactivity disorder"[tiab] OR "attention deficit hyperactivity disorder"[tiab] ) AND ("1900/01/01"[dp] : "2026/08/28"[dp])

## S3 Feeding / ARFID / psychosocial

( ( "food allergy"[tiab] OR "food allergies"[tiab] OR "Food Hypersensitivity"[Mesh] ) AND ( ARFID[tiab] OR "avoidant/restrictive food intake disorder"[tiab] OR "selective eating"[tiab] OR "picky eating"[tiab] OR "feeding disorder"[tiab] OR psychosocial[tiab] OR "mental health"[tiab] OR "behavioral difficulties"[tiab] OR "behavioural difficulties"[tiab] OR "emotional difficulties"[tiab] ) AND ( child*[tiab] OR adolescen*[tiab] OR pediatric*[tiab] OR paediatric*[tiab] ) ) OR ( ( ADHD[tiab] OR "attention-deficit/hyperactivity disorder"[tiab] ) AND ( ARFID[tiab] OR "avoidant/restrictive food intake disorder"[tiab] OR "selective eating"[tiab] OR "picky eating"[tiab] OR "sensory sensitivity"[tiab] ) AND ( child*[tiab] OR adolescen*[tiab] OR pediatric*[tiab] OR paediatric*[tiab] ) ) AND ("1900/01/01"[dp] : "2026/08/28"[dp])

## S4 Microbiota / immune / brain mechanism

( ( "Food Hypersensitivity"[Mesh] OR "food allergy"[tiab] OR "food allergies"[tiab] ) AND ( microbiome[tiab] OR microbiota[tiab] OR "gut microbiota"[tiab] OR "gut microbiome"[tiab] OR metabolite*[tiab] ) AND ( neurobehavior*[tiab] OR behaviour*[tiab] OR behavior*[tiab] OR attention[tiab] OR brain[tiab] OR cognition[tiab] ) ) OR ( ( ADHD[tiab] OR "attention-deficit/hyperactivity disorder"[tiab] ) AND ( microbiome[tiab] OR microbiota[tiab] OR "gut microbiota"[tiab] OR "gut microbiome"[tiab] ) AND ( "systematic review"[tiab] OR "meta-analysis"[tiab] OR "scoping review"[tiab] OR review[pt] ) ) OR ( ( "food allergy"[tiab] OR "food allergies"[tiab] ) AND ( microbiome[tiab] OR microbiota[tiab] OR "gut microbiota"[tiab] OR "gut microbiome"[tiab] ) AND ( "systematic review"[tiab] OR "scoping review"[tiab] OR review[pt] ) AND ( child*[tiab] OR pediatric*[tiab] OR paediatric*[tiab] ) ) AND ("1900/01/01"[dp] : "2026/08/28"[dp])

## S5 FA diagnosis / nutrition / management

( ( "food allergy"[tiab] OR "food allergies"[tiab] OR "IgE-mediated food allergy"[tiab] OR "Food Hypersensitivity"[Mesh] ) AND ( guideline[pt] OR "Practice Guideline"[pt] OR guideline*[ti] OR consensus[ti] ) ) OR ( ( "food allergy"[ti] OR "food allergies"[ti] OR "cow's milk allergy"[ti] ) AND ( nutrition*[ti] OR "nutritional management"[tiab] OR "dietary management"[tiab] OR "oral food challenge"[tiab] ) AND ( review[pt] OR guideline[pt] OR "Practice Guideline"[pt] OR consensus[tiab] ) ) AND ("1900/01/01"[dp] : "2026/08/28"[dp])

## S6 ADHD dietary treatment / probiotics / guideline

( ( ADHD[tiab] OR "attention-deficit/hyperactivity disorder"[tiab] OR "Attention Deficit Disorder with Hyperactivity"[Mesh] ) AND ( "elimination diet"[tiab] OR "few-foods diet"[tiab] OR "few foods diet"[tiab] OR "restricted elimination diet"[tiab] OR "healthy diet"[tiab] OR "food color"[tiab] OR "food colors"[tiab] OR "food colour"[tiab] OR "food colours"[tiab] OR probiotic*[tiab] ) AND ( randomized[tiab] OR randomised[tiab] OR trial[tiab] OR "systematic review"[tiab] OR "meta-analysis"[tiab] OR meta-analysis[pt] OR review[pt] ) ) OR ( ( ADHD[tiab] OR "attention-deficit/hyperactivity disorder"[tiab] ) AND ( guideline[pt] OR "Practice Guideline"[pt] OR guideline*[ti] OR "umbrella review"[tiab] ) ) AND ("1900/01/01"[dp] : "2026/08/28"[dp])
